# Supplementary figures and images for: Predictive Value of Flow Cytometry Quantification of BAL Lymphocytes and Neutrophils in ILD
Source: Cells. 2024 Dec 13;13(24):2066. doi: 10.3390/cells13242066 (PMC11674578; doi:10.3390/cells13242066)

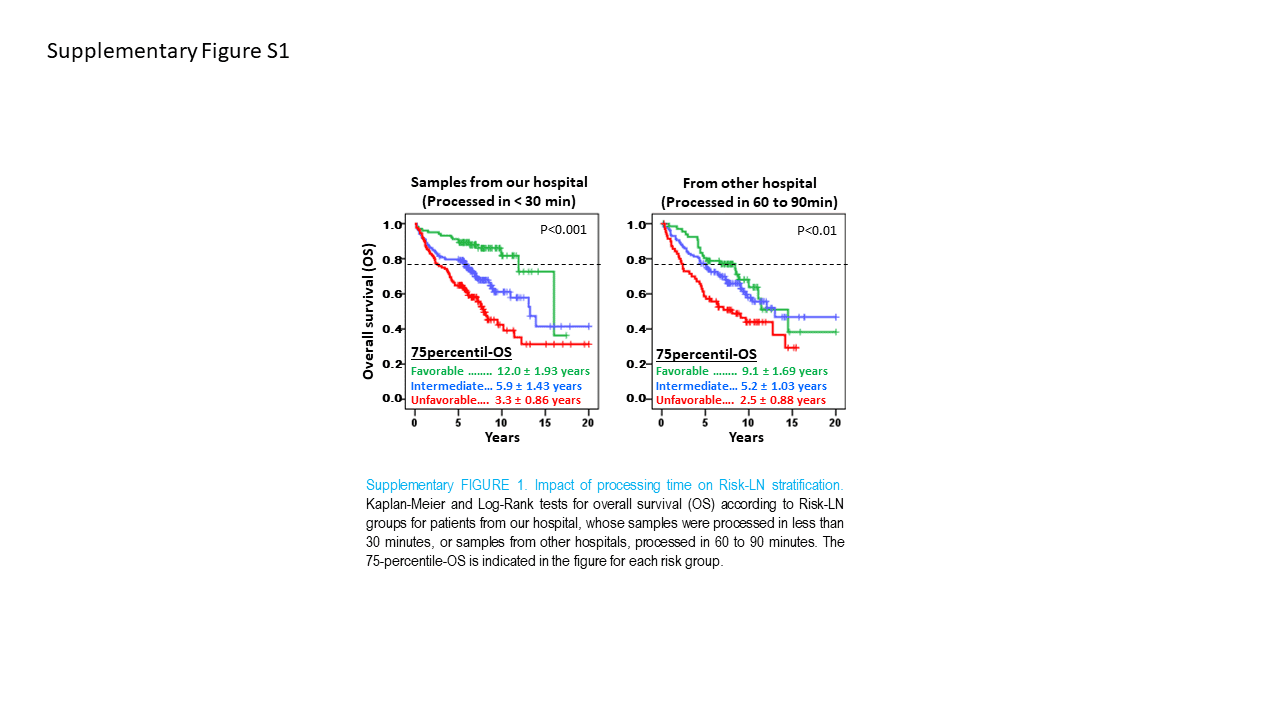

Supplement: Supplementary file 1 [file cells-13-02066-s001.zip › Supplementary Figure S1.png]

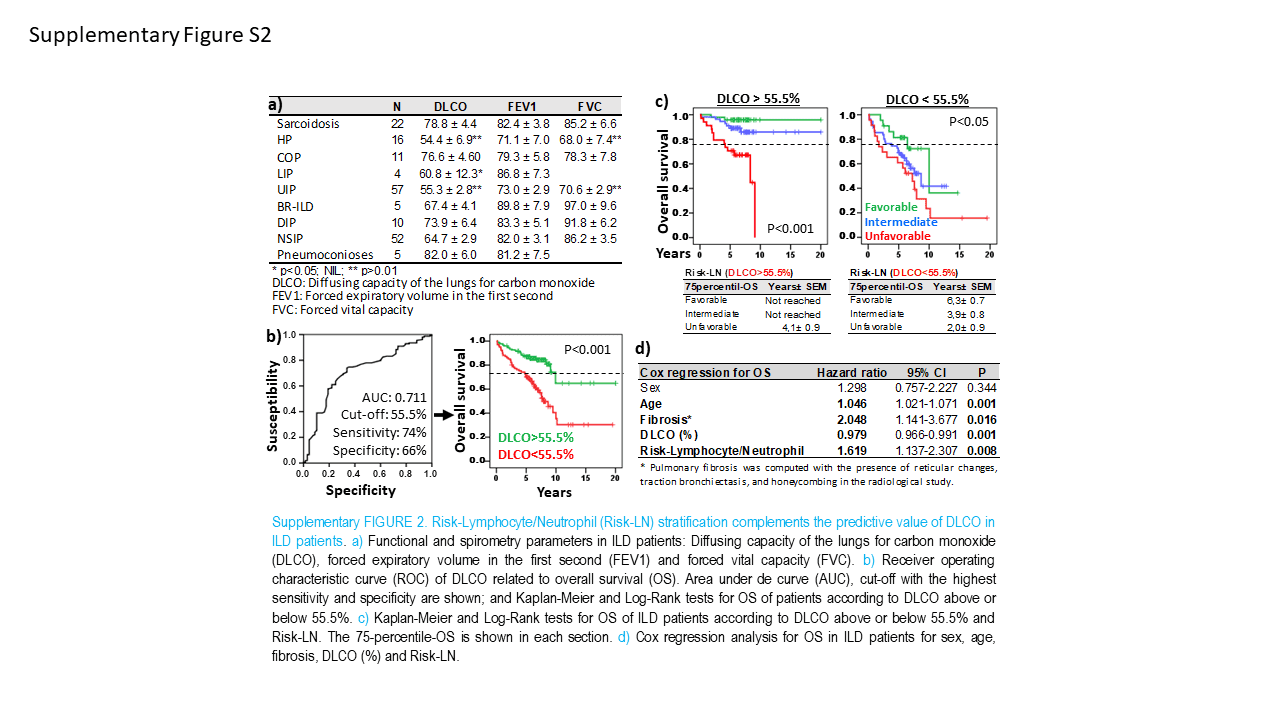

Supplement: Supplementary file 1 [file cells-13-02066-s001.zip › Supplementary Figure S2.png]

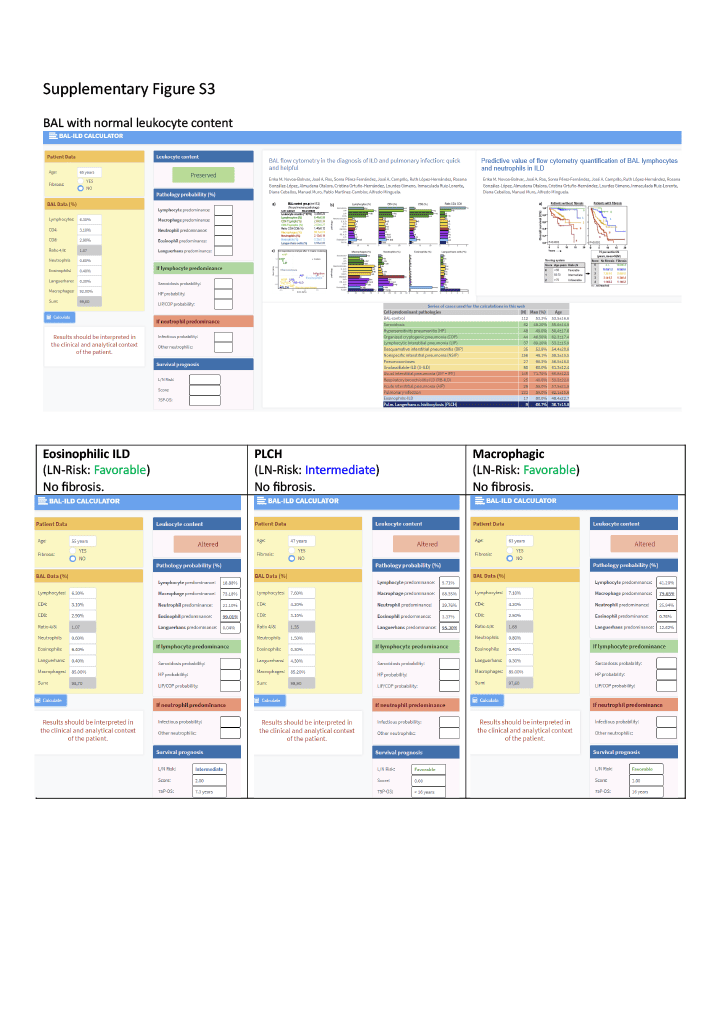

Supplement: Supplementary file 1 [file cells-13-02066-s001.zip › Supplementary Figure S3 P1.png]

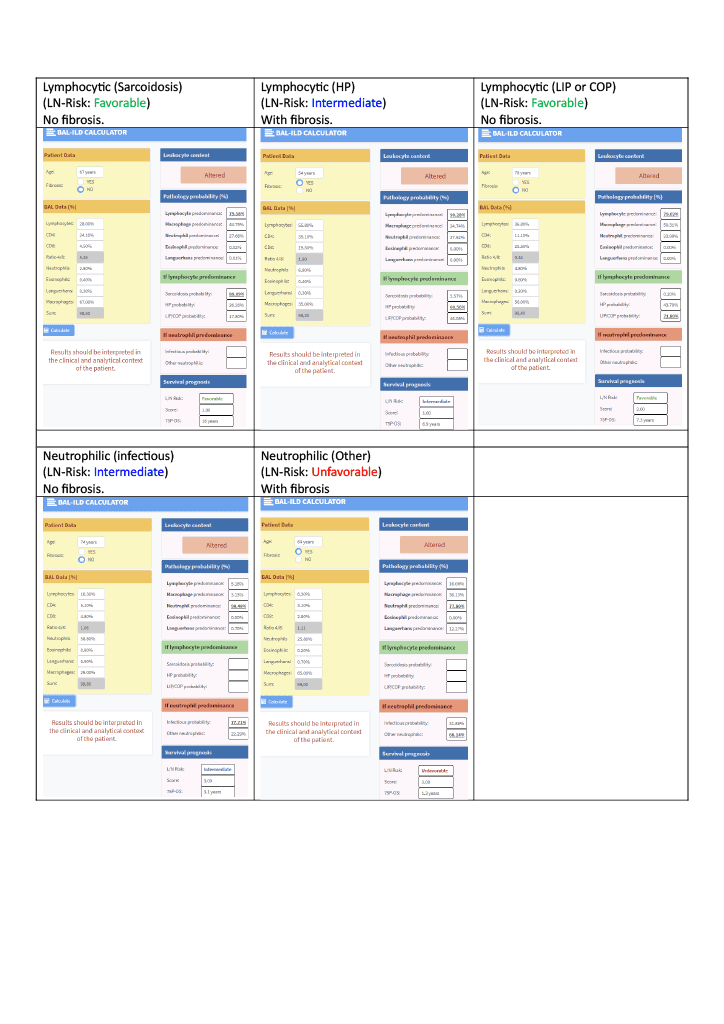

Supplement: Supplementary file 1 [file cells-13-02066-s001.zip › Supplementary Figure S3 P2.png]
